# Determinants of successful guideline implementation: a national cross-sectional survey

**DOI:** 10.1186/s12911-020-01382-w

**Published:** 2021-01-14

**Authors:** Ying-hui Jin, Li-Ming Tan, Khalid S. Khan, Tong Deng, Chao Huang, Fei Han, Jing Zhang, Qiao Huang, Di Huang, Dan-qi Wang, Yu Wang, Xian-tao Zeng, Qiang Wang, Xing-huan Wang

**Affiliations:** 1 Center for Evidence-Based and Translational Medicine, Zhongnan Hospital of Wuhan University, No. 169, Donghu Road, Wuchang District, Wuhan, 430071 China; 2 The Second People’s Hospital of Huaihua, Wuxi Road, Hecheng District, Huaihua, 418200 Hunan China; 3 Women’s Health Research Unit, Queen Mary University of London, Centre for Primary Care and Public Health, Barts and the London School of Medicine, London, UK; 4 Department of General Surgery, Huaihe Hospital of Henan University, No. 8, Baobei Road, Gulou District, Kaifeng, 475000 China; 5 National Center for Medical Service Administration, National Health and Family Planning Commission, Beijing, 100044 China; 6 Emergency Department, Xuan Wu Traditional Chinese Medicine Hospital, Beijing, No. 8, Wanming Road, Xicheng District, Beijing, 10000 China

**Keywords:** Guideline, Adherence, Implementation, Barriers

## Abstract

**Background:**

CPGs are not uniformly successful in improving care and several instances of implementation failure have been reported. Performing a comprehensive assessment of the barriers and enablers is key to developing an informed implementation strategy. Our objective was to investigate determinants of guideline implementation and explore associations of self-reported adherence to guidelines with characteristics of participants in China.

**Methods:**

This is a cross-sectional survey, using multi-stage stratified typical sampling based on China's economic regional divisions (the East, the Middle, the West and the Northeast). 2–5 provinces were selected from each region. 2–3 cities were selected in each province, and secondary and tertiary hospitals from each city were included. We developed a questionnaire underpinned by recommended methods for the design and conduct of self-administered surveys and based on conceptual framework of guideline use, in-depth related literature analysis, guideline development manuals, related behavior change theory. Finally, multivariate analyses were performed using logistic regression to produce adjusted odds ratios (OR) and 95% confidence intervals (95% CI).

**Results:**

The questionnaire consisted of four sections: knowledge of methodology for developing guidelines; barriers to accessing guideline; barriers to guideline implementation; and methods for improving guideline implementation. There were 1732 participants (87.3% response rate) from 51 hospitals. Of these, 77.2% reported to have used guidelines frequently or very frequently. The key barriers to guideline use were lack of education or training (46.2%), and overly simplistic wording or overly broad scope of recommendations (43.8%). Level of adherence to guidelines was associated with geographical regions (the northeast *P* < 0.001; the west *P* = 0.02; the middle *P* < 0.001 compared with the east), hospital grades (*P* = 0.028), length of practitioners’ practice (*P* = 0.006), education background (Ph.D., *P* = 0.027; Master, *P* = 0.002), evidence-based medicine skills acquired in work unit (*P* = 0.012), and medical specialty of practitioner (General Practice, *P* = 0.006; Surgery, *P* = 0.043).

**Conclusion:**

Despite general acknowledgement of the importance of guidelines, the use of guidelines was not as frequent as might have been expected. To optimize the likelihood of adherence to guidelines, guideline implementation should follow an actively developed dissemination plan incorporating features associated with adherence in our study.

## Background

Health research, practice, and policy focus on improving delivery, organization, and outcomes of care. Clinical Practice Guidelines (CPGs) that collate evidence-based recommendations for physicians and other health professionals are critical in this regard [1, 2], and the number of guidelines being published are increasing annually [3]. Guideline implementation, a complex and challenging task [4], requires a change in clinician behavior [5]. Poor implementation may lead to suboptimal patient outcomes as it may miss out on beneficial therapies and may fail to avoid preventable harm, while wasting limited health care resources [4].

In 2010, a Cochrane systematic review by Shaw et al. indicated that guideline implementation interventions selected and tailored according to the prior identification of potential barriers to guideline use were more likely to improve professional practice compared with either no intervention or the dissemination of guidelines alone [6]. It explicitly describes how barriers were identified and how overcoming those barriers could form part of any implementation strategy [6]. In 2013, Flottorp et al. conducted a systematic review of frameworks of determinants of practice and described seven domains of potential determinants of practice: guideline factors, individual health professional factors, patient factors, professional interactions, incentives and resources, capacity for organizational change, and social, political, and legal factors [7]. The Cochrane Effective Practice and Organization of Care (EPOC) group has published a summary of 44 systematic reviews of implementation interventions, giving an indication of the most effective approaches, such as audit and feedback, local consensus conferences, patient-mediated interventions and education outreach [8]. Factors intrinsic to the guideline can also contribute to implementation failure, e.g., ambiguity, inconsistency, and incompleteness, and some guidelines have been found to be more difficult to put into practice than others [9].

An effective implementation strategy involves inclusion of stakeholders in guideline development, identification and overcoming of barriers by assessing individual and organizational preparedness, and capturing the adherence of guidelines via audit and feedback [6–8].

269 guidelines were produced by 256 Chinese developers and 115 were published in Chinese medical journals between 1993 and 2010, yet no systematic and national studies have evaluated strategies that examine barriers and factor related to adherence [10]. Recognizing this information gap, we have investigated the determinants of their implementation and explored the association between guideline adherence and survey participant characteristics in a nationwide study.

## Methods

This study was approved by the Committee for Ethical Affairs of Zhongnan Hospital of Wuhan University.

After having obtained ethics approval, we designed and conducted a robust survey study between January 2019 and July 2019 which complies with recommended methods [11] and aims to maximize compliance with reporting guidelines [12].

### Framework

A multitude of factors, including enablers and barriers of guideline adherence, clinician, organization, and system levels, may influence whether and how guidelines are used [7–9, 13–16]. In order to formally assess the determinants of guideline implementation, we used the following literature and resources for the conceptualization of our research framework: (a) ideas of implementability formalized by Gagliardi et al. [16] consisting of 22 elements within eight domains, including adaptability, usability, applicability, validity, accommodation, communicability, implementation, and evaluation; (b) Guideline Implementability Appraisal (GLIA and GLIA 2.0) tool to provide information about implementability to authoring groups enabling them to decide on content in anticipation of potential problems in implementation [17] and taking into account decidability, executability, validity, flexibility, measurability, effect on process of care, novelty/innovation, and computability [5]; (c) Qualitative approach to exploring the medical practitioners’ perceptions and experiences regarding guideline implementation with general, open-ended and non-leading questions having developed a basic understanding of the reaction of medical practitioners and system mechanism to the introduction of guidelines [18]; (d) Systematic reviews of guideline implementation [19–21] literature with five main areas identified: (1) the guideline, (2) the target health care professional user, (3) the patient characteristics, (4) the work environment, and (5) the implementation strategy; (e) Systematic examination of the content of guideline development manuals to identify implementation methodology of known organizations [22–24]; and (f) Behavior change and social-cognitive theory applied in implementation research for improving understanding of determinants of evidence-based medicine (EBM) practice and guideline use [25–27].

### Item selection

Items collected during the review of literature and existing instruments or frameworks formed the basis of an item pool. This was then further extended with items emerging from interviews with medical practitioner and discussed based on expert opinions and behavior change and social-cognitive theory. Parsimony was achieved by combining multiple items into one, and the number of items was reduced by several expert meetings. The first version was field tested in single interviews among clinicians from Zhongnan Hospital of Wuhan University. A series of draft versions were piloted.

### Questionnaire construction, piloting and reliability testing

A bespoke questionnaire, consisting of four parts, was developed as a self-administered survey and its design was based on recommended methods [11]. The aim of the survey was to investigate barriers and enablers related to guideline adherence. First, the survey instrument covered background information about the participants (qualifications, education level, clinical department, years of practice) and some specific questions related to guideline implementation (e.g., “Have you had EBM or EBM related education?”, “Do you agree that high-quality guidelines provide basic guidance for healthcare delivery?”, “Are you are willing to acquire and read high quality guidelines?”, and “To what extent do you think you are applying the guidelines in your clinical practice?”). A 4-point Likert-type scale was used to rate the extent of guideline adherence.

The second section captured knowledge of a broad and comprehensive range of the methods and processes for producing guidelines with 17 items. Questionnaire items were based on the manuals considered in our framework section (e) as mentioned above, with particular reference to NICE and WHO [22, 23].

Given that guideline noncompliance may come from difficulty in searching or downloading guidelines which is a separate issue to guideline implementation barriers, like guideline flaw, lack of atmosphere of EBP etc., we divided the third section of barriers into guideline acquisition barriers and guideline implementation barriers. So the third section had four multiple choice items relating to barriers to guideline acquisition and 15 multiple choice items relating to barriers to guideline implementation which were categorized into three areas: intrinsic flaw in guideline (eight items); deficient or incomplete system mechanism and external environment (four items); and awareness and ability of clinicians (three items). The fourth part consisted of questions which looked at methods for improving guideline implementation; this included seven multiple choice items which addressed external enablers and four multiple choice items which adopted a microcosmic perspective to focus on internal enablers relating to the guideline implementation. The full questionnaire is provided in Additional file 1.

The content validity and readability of the questionnaire were tested by five experts of guideline development, six clinical EBM experts and 20 clinical experts from different medical specialties. All experts commented on the clarity and relevance of each survey item. There was agreement among the experts on the clarity and relevance of most of the included items, and we revised some items to improve clarity. Before implementing the study survey, we tested for repeatability by administering the questionnaires to the same population of 40 participants twice, with a two-week interval in between the first and second survey. The test–retest reliability coefficient was excellent at 0.80 (1 = perfect repeatability).

### Survey sampling, questionnaire administration and data collection

A cross-sectional survey was used which took into account the differences of geographical location and the number of medical institutions. We used a multi-stage stratified sampling strategy based on China's economic regions (the East with seven provinces and three municipalities; the Middle with six provinces; the West with 11 provinces and one municipality; and the Northeast with three provinces). Two to five provinces were selected for each region with two to three cities selected for every province, and each city included both secondary and tertiary hospitals. Sampling procedures for hospitals was decided by Medical Standards Bureau of Management Center of Medical Management Services, National Health Commission of the People's Republic of China Mainland based on Proportion Report of Hospital Institutions of Health Statistics Yearbook 2018 [28]. We did not include Hong Kong, Macao or Taiwan in this survey. In total, 32 cities from three provinces and two municipalities in the East, three provinces in the Middle, three provinces and one municipality in the West, and two provinces in the Northeast were chosen. More provinces and municipalities were selected from the East because there is a higher concentration of medical institutions in that region. Doctors in each hospital were recruited using the hospitals’ directories which held a database of their ID numbers. Licensed doctors, pharmacists and nurses, regardless of specialty, with over 5 years of continuous working experience of providing direct or indirect clinical care optimizing health promotion, wellness, and disease prevention were invited to take part in the survey.

The survey was administered during the period from January 2019 to July 2019. Four researchers were each allocated to one of the regions and all used the agreed set of instructions included in the protocol. The researcher explained the nature and purpose of the study to the participants in a meeting room. Informed consent was obtained before the printed copies of questionnaires were distributed. The survey data were anonymized. Data were validated using Epidata (version 3.1, Odense Denmark, EpiData Association, 2010). Questionnaires with more than 10% of data missing were excluded from the analysis.

### Data analysis

We hypothesized that the guidelines use is associated with demographic characteristics, attitudes, and knowledge. All included data were analyzed using SPSS [version 17.0 (SPSS, Chicago, IL, USA)]. Categorical variables from survey items were described using frequencies and percentages. Continuous variables were described as median with interquartile range (IQR 25–75% percentile) or mean with standard deviation (SD) as appropriate. We used Chi-squared test to explore if there were differences in the barriers to guideline implementation based on the different grade of hospitals.

Univariate and multivariate analyses were carried out using logistic regression. The dependent variable was the self-reported guideline adherence, and the independent variables were region, hospital grade, years of practice, professional title, EBM education in work unit, education background, EBM education in college, participation in guideline development, acknowledgment of guideline for clinical practice, knowledge score, and professional practice area. Factors of *P* < 0.1 in the univariate analysis were included into the multivariate analysis to identify the independent determinants of guideline adherence. The associations are reported as adjusted odds ratios (ORs) with 95% confidence interval (95% CI). Two-sided *P* < 0.05 was considered to be statistically significant.

## Results

**Fig. 1:**
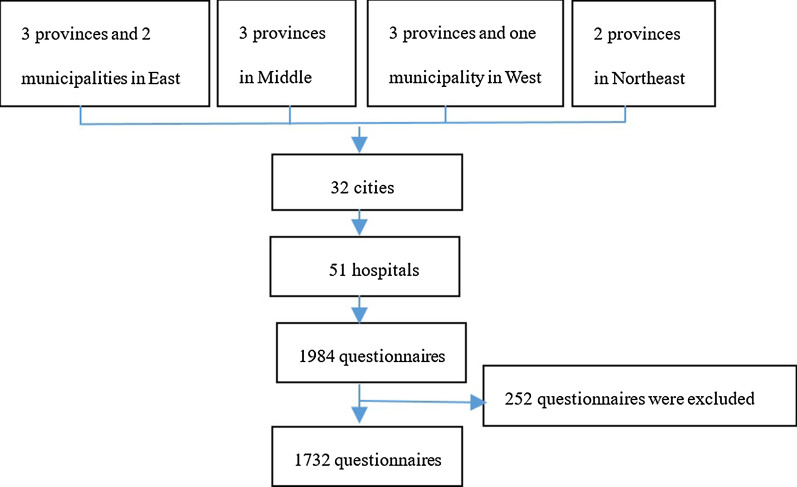
Flow chart of distribution and collection of questionnaires

In total, 1984 questionnaires were administered in 51 hospitals (30 tertiary public hospitals and 21 secondary public hospitals) located in all the main cities in 11 provinces and three municipalities in China (Fig. 1). Of these, 252 questionnaires were excluded because of missing answers (> 10% of data missing). The overall response rate was 87.3% (n = 1732/1984; 1234 and 498 in tertiary and secondary hospitals respectively). As shown in Table 1 the respondents included staff from a wide range of specialties. The specialties “Medicine” and “Surgery” represented 22% and 15.7% of the survey sample, respectively. The median years of participants’ practice was 15.0 (± 10.3) years. Although more than half of participants (54.3%) had received EBM or related education, only a small proportion had participated in the development of guidelines (14.7%). Nearly all participants considered guidelines to provide essential or basic guidance for healthcare delivery (Table 1).

**Table 1:** Characteristics of survey participants

Characteristic	Category	n (%)
Region	The East	710 (41.0%)
	The Middle	361 (20.8%)
	The West	384 (22.2%)
	The Northeast	277 (16.0%)
Grade of hospitals	Tertiary hospital	498 (28.8%)
	Secondary hospital	1234 (71.2%)
Professional practice area	Oncology (including hemolymph neoplasm)	93 (5.7%)
	Stomatology/ophthalmology/otorhinolaryngology	85 (5.2%)
	Medicine	360 (22.0%)
	Surgery	256 (15.7)
	ICU or emergency	62 (3.8%)
	Anesthesia	72 (4.4%)
	Gynaecology/obstetrics	39 (2.4%)
	Traditional Chinese medicine	95 (5.8%)
	Pediatrics	108 (6.6%)
	Clinical pharmacy	157 (9.6%)
	Radiography or medical imaging	81 (5.0%)
	General practice or comprehensive health care	145 (8.9%)
	Nursing	83 (5.1%)
Years of practice		15.0 ± 10.3
Education background	PHD’s	180 (10.6%)
	Master’s	419 (24.7%)
	Bachelor’s	893 (52.5%)
	Junior college	208 (12.2%)
Professional title	Chief physician or professor of medicine	264 (16.5%)
	Associate senior doctor or associate chief physician or associate professor	369 (23.1%)
	Intermediate	526 (32.9%)
	Primary	440 (27.5%)
Have you ever received any EBM or related education in college	Yes	894 (54.3%)
	No	754 (45.8%)
Have you ever received any EBM or related education in work unit	Yes	1210 (73.1%)
	No	445 (26.9%)
Do you think high-quality guidelines provide basic guidance for clinical practice	Yes	1672 (97.4%)
	No	44 (2.6%)
Willing to acquire and study high quality guideline	Yes	1687 (98.2%)
	No	31 (1.8%)
Self-reported guideline adherence	Seldom	50 (2.9%)
	Sometimes	339 (19.9%)
	Frequently	1127 (66.2%)
	Very frequently	186 (10.9%)
Kind of guidelines used	Foreign guideline	199 (12.2%)
	Translated version from foreign guideline	425 (26.0%)
	Chinese version	1009 (61.8%)
Have you ever participated in the development of guidelines	Yes	251 (14.7%)
	No	1458 (85.3%)
If so, what role of participating in guideline development	Chairman	12 (11.0%)
	Final reviewer	33 (30.3%)
	Developer	36 (33.0%)
	Other	28 (25.7%)

### Knowledge for CPGs development

Most of the respondents (94.5%, range 85.4–98.2%) agreed or strongly agreed with all methodological items. Out of all the items considered to be an important component of the key methodology in the development of guidelines, “conducting a systematic and comprehensive search for evidence” was the item that was strongly agreed or agreed upon by the highest proportion of the surveyed population (98.2%) (Additional file 2).

### Barriers and enablers of guideline adherence

Overall, 1313 (77.1%) participants reported frequent or very frequent use of guidelines (61.8% participants were using Chinese guidelines). Only 50 (2.9%) participants seldom used guidelines even though they were aware of the guidelines (Table 1). Table 2 shows barriers to acquisition and implementation of guidelines. A noteworthy finding with regards to the acquisition of guidelines was that over half of the participants were too busy to pursue acquisition (58.6%). The most frequent barrier in implementation of guidelines was “lack of education or training in guideline use” which comes under “awareness and ability of clinicians” domain in our framework, as reported by 787 participants (46.2%). The other most cited implementation barriers were that the “wording of recommendations were too simple or that the scope of the recommendations were too broad”, as reported by 746 participants (43.8%), “lack of agreement between different guidelines dealing with a similar topic” as reported by 699 participants (41.1%), “ambiguity and lack of clarity of recommendations” as reported by 697 participants (41.0%) and “lack of evidence from Chinese sample” as reported by 654 participants (38.4%) which all come under “existing intrinsic flaw of guideline” domain in our framework.

**Table 2:** Barriers to guideline acquisition and implementation

Barriers	n (%)
*Acquisition*	
So busy with work, no time to search for guidelines	992 (58.6)
Limited knowledge of searching for guidelines	631 (37.1)
Less convenient to search for or download foreign language guidelines	894 (52.5)
Difficulty in searching for high quality guidelines	516 (30.4)
*Implementation*	
Wording too simple or recommendations too broad to solve the patient’s practical problem	746 (43.8)
Ambiguity and lack of clarity of recommendations	697 (41.0)
Methods of rating of evidence or recommendations too complex to understand	592 (34.8)
Lack of evidence from Chinese sample	654 (38.4)
Low quality of underlying evidence	365 (21.4)
Lack of agreement between different guidelines dealing with a similar topic	699 (41.1)
Guidelines deemed impractical for use in local setting due to resource factors, such as lack of staff, materials or funding	605 (35.5)
Guideline implementation affects physician’s income	153 (9.0)
Language barriers associated with international guidelines	638 (37.5)
Delayed updates	398 (23.4)
Worry about legal issues because of conflict with usual practice	513 (30.1)
Lack of validity, due to high possibility of the existence of conflict of interest	272 (16.0)
Guideline use is unnecessary, because three level ward-round system can safeguard medical treatment quality	123 (7.2)
Lack of education or training in guideline use	787 (46.2)
Lack of atmosphere to encourage guideline use, for example lack of support from leaders or no culture of EBM	320 (18.8)

**Fig. 2:**
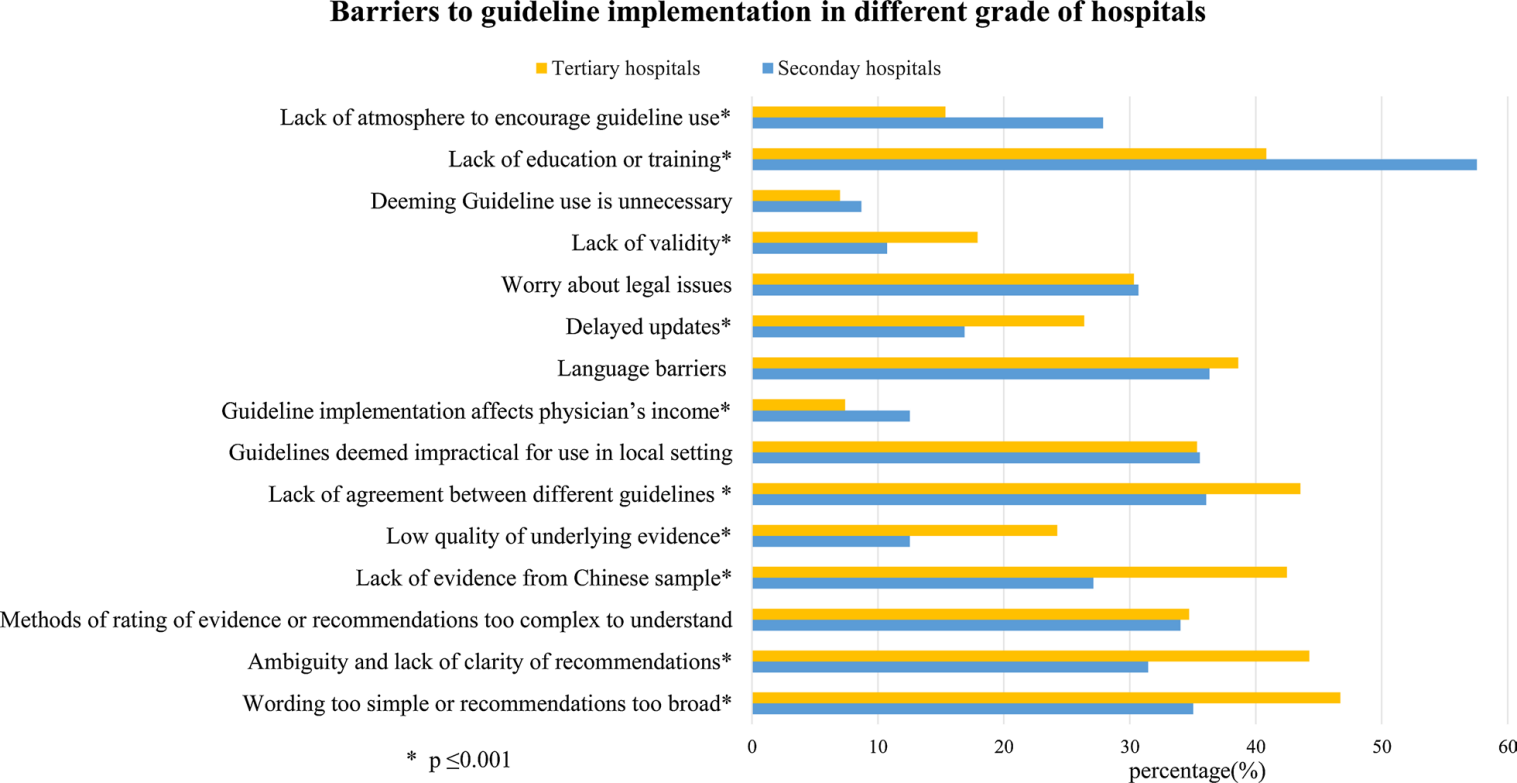
Barriers to guideline implementation in secondary versus tertiary hospitals

When compared to the answers provided by participants in tertiary hospitals, more health care practitioners in secondary hospitals thought that the lack of a conducive atmosphere to encourage guideline use (*P* < 0.001), lack of education or training (*P* < 0.001), guideline implementation affects physician’s income (*P* < 0.001) were barriers of guideline use (Fig. 2).

When compared to the answers provided by participants from secondary hospitals, more health care practitioners in tertiary hospitals thought that the lack of validity (*P* < 0.001), delayed updates (*P* < 0.001), lack of agreement between different guidelines(*P* < 0.001), low quality of underlying evidence (*P* < 0.001), lack of evidence from Chinese sample (*P* = 0.001), ambiguity and lack of clarity of recommendations (*P* < 0.001) and overly simplistic wording or overly broad scope of recommendations (*P* < 0.001) were barriers to guideline implementation (Fig. 2).

Additional file 3 showed data on enablers and we found that the utilization of various media, short format presentations, linking of guidelines to patient electronic medical records, identification of the possible barriers, facilitators, or feasible solutions, and provision are important guideline implementation tools.

### Features associated with guideline adherence

Multivariate analysis showed that adherence was associated with regions (Northeast OR 2.02, 95% CI 1.42–2.88, *P* < 0.001; West OR 1.53, 95% CI 1.07–2.18, *P* = 0.02; Middle OR 2.16, 95% CI 1.48–3.15, *P* < 0.001 compared with the East), hospital grades (OR 0.70, 95% CI 0.50–0.96, *P* = 0.028), practitioners’ years of practice (OR 1.03, 95% CI 1.01–1.05, *P* = 0.006), education background (PhD OR 1.90, 95% CI 1.07–3.37, *P* = 0.027; Master OR 2.11, 95% CI 1.31–3.41, *P* = 0.002 compared with Junior college degree), EBM skills acquired in work unit (OR 1.44, 95% CI 1.08–1.92, *P* = 0.012), and specialty (General practice OR 2.06, 95% CI 1.23–3.45, *P* = 0.006; Surgery OR 1.49, 95% CI 1.01–2.19, *P* = 0.043). Participants in the Northeast, Middle, and West regions were more likely than those in the East region to consider using guidelines. Participants in secondary public hospitals showed higher self-reported guideline adherence than those in tertiary public hospitals. The longer the years of practice and the higher the education background, the more likely participants were to considered guideline use. We found that EBM or EBM-related education in work unit is significantly associated with self-reported guideline adherence.

No associations were found between professional titles (chief physician or professor of medicine, associate senior doctor or associate chief physician), EBM or related education in college, acknowledgment of guidelines providing basic guidance for clinical practice, participation in the development of guidelines, and knowledge scores for guideline development (Table 3).

**Table 3:** Determinants associated with guidelines adherence

Variables	Univariate logistic regression		Multivariate logistic regression	
	OR (95% CI)	*p* value	OR (95% CI)	*p* value
*Region*				
The Northeast	2.60 (1.87,3.60)	< 0.001	2.02 (1.42,2.88)	< 0.001
The West	1.98 (1.46,2.69)	< 0.001	1.53 (1.07,2.18)	0.02
The Middle	2.68 (1.95,3.67)	< 0.001	2.16 (1.48,3.15)	< 0.001
The East	1.000		1.000	
*Hospital grade*				
Tertiary hospital	0.61 (0.47,0.80)	< 0.001	0.70 (0.50,0.96)	0.028
Secondary hospital	1.000		1.000	
Years of practice	1.03 (1.02,1.04)	< 0.001	1.03 (1.01,1.05)	0.006
*Education background*				
PHD’s	1.93 (1.21,3.09)	0.006	1.90 (1.07,3.37)	0.027
Master’s	2.08 (1.39,3.10)	< 0.001	2.11 (1.31,3.41)	0.002
Bachelor’s	1.62 (1.12,2.33)	0.01	1.34 (0.88,2.02)	0.169
Junior college	1.000		1.000	
*Professional title*				
Chief physician or professor of medicine	1.86 (1.32,2.62)	< 0.001	0.68 (0.37,1.24)	0.208
Associate senior doctor or associate chief physician	2.08 (1.53,2.84)	< 0.001	0.94 (0.60,1.47)	0.78
Intermediate	1.57 (1.18,2.08)	0.002	1.01 (0.72,1.43)	0.951
Primary	1.000		1.000	
EBM or EBM related education in college	1.17 (0.94,1.47)	0.166		
EBM or EBM related education in work unit	1.54 (1.19,1.99)	0.001	1.44 (1.08,1.92)	0.012
Participation in the development of guidelines	0.77 (0.56,1.05)	0.101		
Acknowledgment of guideline providing basic guidance for clinical practice	4.01 (2.04,7.86)	< 0.001	2.10 (0.98,4.50)	0.055
Knowledge scores for guideline development	1.00 (0.99,1.02)	0.887		
*Professional practice area*				
ICU or emergency	1.23 (0.74,2.04)	0.433	1.05 (0.61,1.82)	0.848
Pediatrics	1.75 (1.08,2.82)	0.023	0.97 (0.56,1.70)	0.908
Gynaecology/obstetrics	1.68 (0.88,3.19)	0.115	1.10 (0.56,2.17)	0.774
Nursing	2.03 (1.14,3.66)	0.016	1.74 (0.94,3.23)	0.077
Stomatology/ophthalmology/otorhinolaryngology	0.74 (0.44,1.23)	0.244	0.88 (0.51,1.52)	0.639
Anesthesia	1.66 (0.90,3.05)	0.106	1.27 (0.66,2.45)	0.483
General practice or comprehensive health care	2.64 (1.66,4.19)	< 0.001	2.06 (1.23,3.45)	0.006
Surgery	1.92 (1.33,2.76)	< 0.001	1.49 (1.01,2.19)	0.043
Oncology	2.13 (1.25,3.60)	0.005	1.47 (0.82,2.64)	0.191
Clinical pharmacy	0.64 (0.43,0.96)	0.03	0.74 (0.48,1.15)	0.18
Radiography or medical imaging	1.50 (0.83,2.70)	0.179	1.25 (0.67,2.33)	0.486
Traditional Chinese medicine	1.77 (0.80,3.90)	0.157	1.64 (0.68,3.98)	0.271
Medicine	1.000		1.000	

*CI* confidence interval, *OR* odds ratio

## Discussion

### Main findings

Our survey found that over two-thirds of practitioners used guidelines frequently or very frequently and had a positive attitude towards the guidelines’ potential impact on their clinical practice. The key barriers to guideline use were lack of education or training, overly simplistic wording of recommendations or overly broad scope of recommendations, and disagreement between guidelines on the same topic. Secondary hospitals showed higher adherence than tertiary hospitals. Guideline adherence was associated with regions, hospital grades, practitioners’ years of practice, education background, EBM skills acquired in work unit, and general practice or surgical specialty.

### Strengths and limitations

To our knowledge this is the first nationally representative survey that used a reliable and validated instrument to examine the factors influencing guideline implementation among Chinese health care practitioners. With an excellent response rate and geographical coverage, we believe that our results should be representative of other parts of China. However, as Hong Kong, Macao and Taiwan were omitted from the survey, and that we did not include first level hospitals and grass-roots medical and health institutions, these limitations should be borne in mind with respect to generalizability. Furthermore, the cross-sectional design limits inferences concerning causal relationships. Finally, as in all self-reported data, social desirability bias may lead to an overestimation of guideline use. Overall, we believe that the quality of our data is sufficiently strong for use in the development of implementation strategies that target the identified barriers to guideline implementation.

### Interpretation of findings

Nothing could be more frustrating to a guideline developer than failure of its implementation. Neither the positive attitude towards the significance of guideline implementation nor the knowledge of key methodology for developing guidelines were the prerequisite for guideline use. The result is similar to a survey by Mengyu Liu, et al. in China. They investigated the views of Chinese medicine (CM) doctors on guidelines in China, and showed that a majority of respondents stated that they were familiar with CPGs, however, significantly fewer claimed to be following some form of these [29].

Many factors may influence the implementation of a guideline in China. The most commonly perceived barriers were related to “lack of education or training for guideline use” which comes under “incomplete system mechanism or external environment”. Linan Zeng reported, in primary care settings in China, only 11.3% frequently used CPG, and the most frequently identified barrier to guideline use was lack of training (49.9%) which is consistent with those of our study [30]. A scoping review stated that print material and education for professionals and patients were the most commonly employed strategy for translating guidelines to practice [21]. Workforce education for guideline implementation as an important strategy was illustrated by multiple guideline development manuals [22–24]. Our research also stated that EBM or EBM related education in the work unit is significantly associated with self-reported guideline adherence. So education programs should take those factors into account. Education in guideline use and building an atmosphere to encourage guideline use is more important for lower grade hospitals, since our research showed that more health care practitioners in secondary hospitals chose lack of education and atmosphere to encourage guideline use as barriers of guideline use than tertiary hospitals. Workforce education should be interprofessional in scope and integrated in practice [31]. The major stakeholders, including representatives of the various practitioner and patient groups as well as local administrators and policy makers, should be engaged [22–24, 31].

We found that ambiguity in the wording of the recommendations confused practitioners and hampered uptake of guidelines, as reported previously [32]. Key action statements should be clear so as to prevent inappropriate practice variation [33]. Confidence in ability to practice the recommended behavior is key to its implementation [34–36]. Formulating recommendations and suggestions can be based on the “Who? What? Where? How?” approach suggested by guideline development manuals to standardize the recommendations and wording [37].

Style, content, and format consistency with transparency in rationale and congruence with organization-specific policies can improve implementation [33, 38]. With the development of information technology [39], adapting the form of presentation for mobile devices, pocket guides, wall posters and summary versions are badly needed for just-in-time accessibility.

Guideline implementation plans tailored to overcome the potential barriers identified in advance are more likely to improve healthcare compared with passive dissemination of guidelines [6, 34]. There are many known features that influence the journey of evidence and guidelines from publication into practice [7, 8, 19, 21]. Features intrinsic to guideline development, including stakeholder involvement during all stages from its conception and content development to formatting and dissemination, are now recognized as important components [9, 23]. Responding to the recognized need for understanding why guideline implementation works in some contexts and not in others, our study sheds light directly on this matter through a national representative survey. Francke et al. [19] performed a systematic review and reported that effective strategies usually have multiple elements and that the use of a single strategy is less effective.

Our study found that unimpeded promotion, multichannel and multiform guideline presentation combined with extensive education and training of all health care practitioners were considered paramount for guideline implementation. Concerns about how the perceived need for high level of resource hinders guideline implementation have been addressed by developing a framework for stratifying guideline recommendations according to health care setting level [32].

The National Health Commission could suggest that specialty associations or societies establish specific guideline implementation working groups to participate in guideline development. For example, each guideline expert panel has a Practice Guidelines Implementation Network (PGIN) representative, and their role is to examine whether guideline recommendations can be implemented in real world practice [40].

There is no doubt that Chinese guideline developers will improve guideline recommendations in such a way that they will be more applicable to different health care settings in China in the future.

## Conclusion and implications

Our survey provides a comprehensive, valid and generalizable snapshot to understand the state of guideline implementation in China, with lessons for other countries and regions. Major challenges lie ahead in: (a) making guidelines more accessible at the point of care; (b) strengthening guideline development, focusing on unambiguous presentation of recommendations with bespoke implementation tools; and (c) training medical staff to embrace guidelines. In conclusion, guideline development should tailor the content for effective dissemination and, for optimizing the likelihood of adherence, guideline implementation should follow a bespoke plan incorporating features identified through our study.

## Supplementary information

**Additional file 1.** The survey questionnaire.

**Additional file 2.** Table of Knowledge scores of key methodology for developing guidelines.

**Additional file 3.** Table of Enablers for guideline implementation in all respondents.

## Data Availability

The datasets used and/or analyzed during the current study are available from the corresponding author on reasonable request.

## Abbreviations

CPGs: Clinical Practice Guidelines
GLIA: Guideline Implementability Appraisal
IQR: Interquartile range
SD: Standard deviation
OR: Odds ratios
CI: Confidence interval
