# Supplementary material for: Determinants of successful guideline implementation: a national cross-sectional survey
Source: BMC Med Inform Decis Mak. 2021 Jan 14;21:19. doi: 10.1186/s12911-020-01382-w (PMC7807713; doi:10.1186/s12911-020-01382-w)
Supplement: Supplementary file 1 — Additional file 1. The survey questionnaire. [file 12911_2020_1382_MOESM1_ESM.docx]

**Appendix 1 the full questionnaire**

**1 Demographic information**

1. Province: _______
2. City: _______

(3) Professional practice area (Specialty): _______

(4) Years of practice: _______

(5) Education background: _______

(6) Professional title: _______

(7) Have you ever received any EBM or EBM related education in college?

Yes□ No□

(8) Have you ever received any EBM or EBM related education in [work unit](javascript:;)?

Yes□ No□

(9) Do you think high-quality guidelines provide “basic guidance” for clinical practice?

Yes□ No□

(10) If conditions permit, are you willing to acquire and read high quality guideline?

Yes□ No□

(11) What extent do you think you have been applying the guidelines for clinical practice (Self-reported guideline adherence)?

Very poor(seldom) 1□ 2□ 3□ Very good(very frequently)4□

(12) What kind of guidelines do you use more?

□ Foreign guidelines □ Translation or adaptation from foreign guidelines

□Chinese guidelines

(13) Have you ever participated in the development of guidelines?

Yes□ No□

If so, what was your role in guideline development?

Chairman□ final reviewer□ developer□ other□

**2 Rate your agreement with the following items for knowledge of Key methodological points for developing guidelines**

| **Items** | **Strongly**  **agreed** | **Agree** | **Unsure** | **Disagree** | **Strongly disagree** |
| --- | --- | --- | --- | --- | --- |
| (1) Guidelines should be developed by an [authority](javascript:;), for example [Health Administrative Departments](javascript:;) or [Professional Society](javascript:;) |  |  |  |  |  |
| (2) Guideline should be registered prior to development |  |  |  |  |  |
| (3) Guideline protocol should be made prior to development |  |  |  |  |  |
| (4) All guideline developers should be educated in guideline methodology through a training program |  |  |  |  |  |
| (5) Guideline development groups to include representatives from a range of relevant stakeholder groups, e.g., experts from related specialties, patients, [methodologist](javascript:;) |  |  |  |  |  |
| (6) Declare, manage and report conflict of interests of all developers |  |  |  |  |  |
| (7) Key questions and priority outcomes should be finalized before systematic literatures search |  |  |  |  |  |
| (8) Conduct a systematic and comprehensive search for evidence |  |  |  |  |  |
| (9) Where research evidence is unavailable for the most important question/problem this e can be addressed by expert consensus |  |  |  |  |  |
| (10) Perform the quality assessment of searched evidence |  |  |  |  |  |
| (11) Evidence evaluation criteria or scales have a clear source, & strong operability |  |  |  |  |  |
| (12) Endeavor to identify and evaluate existing systematic reviews or conducting systematic reviews |  |  |  |  |  |
| (13) As well as considering evidence of effectiveness, safety evidence and patient’s values should also be taken into account |  |  |  |  |  |
| (14) There should be clear and specific criteria for rating the quality of evidence and grading the strength of recommendations |  |  |  |  |  |
| (15) Guidelines should report the consensus methods used to reach agreement on recommendations |  |  |  |  |  |
| (16) Before the guideline is published it should be submitted to external review |  |  |  |  |  |
| (17) Guidelines should be updated periodically |  |  |  |  |  |

**3 Barriers to guideline acquisition and guideline implementation**

**(1) Please choose the barriers to guideline acquisition (Select all that apply)**

| **Barriers to Guideline acquisition** | YES | NO |
| --- | --- | --- |
| So busy with work, no time to search for guidelines |  |  |
| Limited knowledge of searching for guidelines |  |  |
| Less convenient to search for or download foreign language guidelines |  |  |
| Difficulty in searching for high quality guidelines |  |  |

**(2) Please choose the barriers to guideline implementation (Select all that apply)**

| **Barriers to guideline implementation** | YES |
| --- | --- |
| (1) Wording too simple or recommendations too broad to solve the patient’s practical problem |  |
| (2) Ambiguity and lack of clarity of recommendations |  |
| (3) Methods of rating of evidence or recommendations too complex to understand |  |
| (4) Lack of evidence from Chinese sample |  |
| (5) Low quality of underlying evidence |  |
| (6) Lack of agreement between different guidelines dealing with a similar topic |  |
| (7) Guidelines deemed impractical for use in local setting due to resource factors, such as lack of staff, materials or funding |  |
| (8) Guideline implementation affects physician’s income |  |
| (9) Language barriers associated with international guidelines |  |
| (10) Delayed updates |  |
| (11) Worry about legal issues because of conflict with usual practice |  |
| (12) Lack of validity, due to high possibility of the existence of conflict of interest |  |
| (13) Use guideline is unnecessary, since three level ward-round system can safeguard medical treatment quality |  |
| (14) Lack of education or training in guideline use |  |
| (15) Lack of atmosphere to encourage guideline use, for example lack of support from leaders or no culture of EBP |  |

**4 Enablers for guideline implementation in all respondents**

| Enablers for guideline implementation | YES | NO |
| --- | --- | --- |
| **Version or form of guideline** | | |
| (1) Short formats presentation |  |  |
| (2) Utilization of various media |  |  |
| (3) Linking to patient electronic medical records |  |  |
| (4) Discourse by guideline developers |  |  |
| (5) Combine with clinical pathway |  |  |
| (6) Support and facilitation of guideline implementation by administrative leaders of health service institutions |  |  |
| (7) Dissemination and promotion of guidelines by government health department, via teaching events (e.g. national conferences, continuing professional education, etc.) |  |  |
| Guideline document | | |
| (1) Identify the possible barriers, facilitators, or feasible solutions needed for specified recommendations |  |  |
| (2) Provide guideline implementation tools (implementation tool means any self-contained informational or interactive print or electronic resources in the guideline document or accompanying files, websites, or applications) |  |  |
| (3) Clarify the equipment, staff or corresponding training needed for implementing recommendation |  |  |
| (4) Provide baseline assessment tool, audit tool& measurement tool |  |  |
