# Supplementary material for: Determinants of successful guideline implementation: a national cross-sectional survey
Source: BMC Med Inform Decis Mak. 2021 Jan 14;21:19. doi: 10.1186/s12911-020-01382-w (PMC7807713; doi:10.1186/s12911-020-01382-w)
Supplement: Supplementary file 2 — Additional file 2. Table of Knowledge scores of key methodology for developing guidelines. [file 12911_2020_1382_MOESM2_ESM.docx]

**Appendix 2 Knowledge scores of key methodology for developing guidelines**

| **Items** | **Strongly**  **agreed** | **Agree** | **Unsure** | **Disagree** | **Strongly disagree** |
| --- | --- | --- | --- | --- | --- |
| Guideline should be developed by an [authority](javascript:;), for example [Health Administrative Departments](javascript:;) or [Professional Society](javascript:;) | 1180(71.7%) | 416(25.3%) | 36(2.2%) | 10(0.6%) | 5(0.3%) |
| Guideline should be registered prior to development | 759(46.6%) | 632(38.8%) | 201(12.3%) | 35(2.2%) | 2(0.1%) |
| Guideline protocol should be made prior to development | 931(58.0%) | 619(38.5%) | 51(3.2%) | 4(0.3%) | 1(0.1%) |
| All guideline developers should be educated in guideline methodology by a training program | 1049(64.79%) | 511(31.56%) | 50(3.09%) | 8(0.5%) | 1(0.1%) |
| Guideline development groups to include representatives from a range of relevant stakeholder groups，e.g.，experts from related specialties, patients, [methodologist](javascript:;) | 1060(65.1%) | 495(30.4%) | 60(3.7%) | 12(0.7%) | 2(0.1%) |
| Declare, manage and report conflict of interests of all developers | 860(52.9%) | 622(38.3%) | 128(7.9%) | 13(0.8%) | 2(0.1%) |
| Key questions and priority outcomes should be finalized before systematic literatures search | 916(56.2%) | 663(40.7%) | 44(2.7%) | 5(0.31%) | 1(0.1%) |
| Conduct a systematic and comprehensive evidence search | 1048(64.7%) | 541(33.4%) | 27(1.7%) | 2(0.1%) | 1(0.1%) |
| Where research evidence is unavailable for the most important question/problem this can be addressed by expert consensus | 842(51.8%) | 673(41.4%) | 105(6.5%) | 4(0.3%) | 2(0.1%) |
| Perform the quality assessment of searched evidence | 1115(68.5%) | 478(29.4%) | 30(1.8%) | 3(0.2%) | 1(0.1%) |
| Evidence evaluation criteria or scales have a clear source &strong operability | 1058(65.1%) | 521(32.0%) | 44(2.7%) | 2(0.1%) | 1(0.1%) |
| Endeavor to identify and evaluate existing systematic reviews or conduct systematic reviews | 811(50.2%) | 686(42.4%) | 112(6.9%) | 7(0.4%) | 1(0.1%) |
| As well as considering evidence of effectiveness,  safety evidence and patients values should also betake into account | 968(59.3%) | 607(37.2%) | 52(3.2%) | 5(0.3%) | 1(0.1%) |
| There should be clear and specific criteria for rating the quality of evidence and grading the strength of recommendations | 940(58.1%) | 599(37.0%) | 72(4.5%) | 5(0.3%) | 1(0.1%) |
| Guideline should report the consensus methods used to reach agreement on recommendations | 822(50.9%) | 622(38.5%) | 160(9.9%) | 10(0.6%) | 1(0.1%) |
| Before the guideline is published it should be submitted to external review | 870(54.0%) | 587(36.4%) | 137(8.5%) | 17(1.1%) | 1(0.1%) |
| [Guidelines should be updated](javascript:;)  [periodically](javascript:;) | 1118(68.8%) | 460(28.3%) | 43(2.6%) | 4(0.3%) | 1(0.1%) |
