# Supplementary material for: Determinants of successful guideline implementation: a national cross-sectional survey
Source: BMC Med Inform Decis Mak. 2021 Jan 14;21:19. doi: 10.1186/s12911-020-01382-w (PMC7807713; doi:10.1186/s12911-020-01382-w)
Supplement: Supplementary file 3 — Additional file 3. Table of Enablers for guideline implementation in all respondents. [file 12911_2020_1382_MOESM3_ESM.docx]

**Appendix 3 Enablers for guideline implementation in all respondents**

| **Items** | **n (% of total)** |
| --- | --- |
| Version or form of guideline | n (% of 1732 total) |
| Short formats presentation | 1402(81.9%) |
| Utilization of various media | 1439(84.1%) |
| Linking to patient electronic medical records | 1015(59.3%) |
| Discourse by guideline developers | 987(57.7%) |
| Combine with clinical pathway | 887(51.8%) |
| Support and facilitation of guideline implementation by administrative leaders of health service institutions | 963(56.3%) |
| Dissemination and promotion of guidelines by government health department, via teaching events (e.g. national conferences, continuing professional education, etc.) | 891(52.1%) |
| **Guideline document** | **n(% of 1700 total)** |
| Identify the possible barriers, facilitators, or feasible solutions needed for specified recommendations | 1223(71.9%) |
| Provide guideline implementation tools (implementation tool means any self-contained informational or interactive print or electronic resources in the guideline document or accompanying files, websites, or applications) | 1110(65.3%) |
| Clarify the equipment, staff or corresponding training needed for implementing recommendation | 1020(60.0%) |
| Provide baseline assessment tool, audit tool& measurement tool | 816(48.1%) |
